# Correction: Orban et al. To Hemoadsorb or Not to Hemoadsorb—Do We Have the Answer Yet? An Updated Meta-Analysis on the Use of CytoSorb in Sepsis and Septic Shock. *Biomedicines* 2025, *13*, 180

**DOI:** 10.3390/biomedicines13071573

**Published:** 2025-06-27

**Authors:** Carmen Orban, Angelica Bratu, Mihaela Agapie, Tudor Borjog, Mugurel Jafal, Romina-Marina Sima, Oana Clementina Dumitrașcu, Mihai Popescu

**Affiliations:** 1 Obstetrics and Gynecology, Anesthesia and Intensive Care, Department 14, School of Medicine, “Carol Davila” University of Medicine and Pharmacy, 37 Dionisie Lupu Street, 020021 Bucharest, Romania; carmen.orban@umfcd.ro (C.O.); agapiemili@yahoo.com (M.A.); tudorborjog@gmail.com (T.B.); mugureljafal@yahoo.com (M.J.); romina.sima@umfcd.ro (R.-M.S.); mihai.popescu@umfcd.ro (M.P.); 2 Bucharest University Emergency Hospital, 169 Splaiul Independentei, 050098 Bucharest, Romania; oanadumit@gmail.com; 3 “Bucur” Maternity, “Saint John” Hospital, 040294 Bucharest, Romania

**Errors in Figure/Table**

In the original publication, there were three mistakes in Table 1 [1]. The characteristics of the included studies and therapies applied, as published. In the study of Hawchar et al. [2], CytoSorb was used as a stand-alone adsorption column and not in conjunction with Continuous Renal Replacement Therapy (CRRT)**.** The study by Schittek et al. [3] is listed as originating from Austria. However, the study was conducted in Germany. Also, the name of the first article, published as reference 18, was missing and has now been added. The corrected Table 1, showing the characteristics of the included studies and therapies applied, appears below. The authors state that the scientific conclusions are unaffected. This correction was approved by the Academic Editor. The original publication has also been updated.

**Text Correction**

There were two errors in the abstract of the original publication. First, there was a transcription error regarding the statistical results in terms of comparing early initiation of CytoSorb-treated patients with controls. The correct statistical results are (OR 2.02, 95% CI [1.06, 3.85], *p* = 0.03). The second error is regarding the conclusion stated in the abstract. According to our statistical analysis, the addition of CytoSorb to CRRT may be associated with an increased survival, not a decreased survival, as compared to CRRT alone. A correction has been made in the abstract.

“Abstract: Severe inflammation leading to organ dysfunction is the cornerstone of the pathophysiology of sepsis. Thus, from a theoretical point of view, rebalancing inflammation has the potential to improve patient outcomes. Methods: To better understand the clinical effectiveness of hemoadsorption in managing inflammation, we conducted an updated meta-analysis on the effects of CytoSorb in critically ill septic patients. Ten studies containing 715 patients (355 in the interventional group and 360 in the control group) have been included in the final analysis. Results: Statistical analysis demonstrated that the use of CytoSorb did not influence overall mortality (OR 0.95, 95% CI [0.58, 1.56], *p* = 0.85), but we observed a decreased mortality when comparing CytoSorb-treated patients with patients in the control group treated with continuous renal replacement therapy (CRRT) (OR 0.97, 95% CI [0.46, 0.98], *p* = 0.04). We also observed an increased mortality in patients in whom hemoadsorption was initiated earlier in the treatment course (OR 2.02, 95% CI [1.06, 3.85], *p* = 0.03). We did not observe any significant difference in either intensive care unit length of stay (*p* = 0.93) or between end-of-treatment severity scores in the two groups (*p* = 0.24). Conclusions: Although it has a high risk of bias, current evidence does not support the routine use of CytoSorb in critically ill septic patients. The addition of CytoSorb to CRRT may be associated with increased survival as compared to CRRT alone, but future studies are needed to draw a definitive conclusion.”

The same errors were made in the results section of the original manuscript. The transcription error regarding the statistical results in terms of comparing early initiation of CytoSorb-treated patients with controls and the conclusion regarding the survival benefits of CytoSorb compared to CRRT alone. A correction has been made in the abstract.

The first correction was made in the results section, Section 3.1. Mortality assessment, paragraph 3.

“Three studies were identified as fulfilling the criteria of the early initiation of CytoSorb and were included in the subgroup analysis. Of these, two were randomized control trials, and one was a case–control matched analysis. Statistical analysis demonstrated increased mortality in the early initiation of CytoSorb treatment (67.9%) compared to the controls (52.3%) (OR 2.02, 95% CI [1.06, 3.85], *p* = 0.03)—Figure 5.”

The second correction was made in the conclusions section.

“Despite not being the “new kid on the block”, current evidence for the use of CytoSorb in patients with sepsis and septic shock does not support its routine use but rather a more personalized approach in patients who have severe systemic inflammation. Although it has a high risk of bias, the current evidence does not support the routine use of CytoSorb in critically ill septic patients. The addition of CytoSorb to CRRT may be associated with increased survival as compared to CRRT alone, but future studies are needed to draw a definitive conclusion.”

The authors state that the scientific conclusions are unaffected. This correction was approved by the Academic Editor. The original publication has also been updated.

## Figures and Tables

**Table 1:** Characteristics of included studies and therapies applied.

Publication	Type of Study	Type of Hospital	Country of Origin	Patients: Total (CS/C)	Age (CS/C)	SOFA/APACHE II Score (CS/C)	Treatment Therapy	Control	Commencement of Therapy	Mortality Outcome (CS/C)
Schadler et al. [2017] [18]	Open-label, RCT	NA	Germany	97 (47/50)	66/65	NR	CytoSorb hemoperfusion for 6 h/day for up to 7 days	Standard medical care	Within 72 h of diagnosis	44.7%/26.0%
Hawchar et al. [2019] [19]	RCT	UH	Hungary	20 (10/10)	60/71	13.6/12.8	24 h stand-alone CytoSorb	Standard medical care	Within 24 h of diagnosis	50.0%/50.0%
Brouwer et al. [2019] [20]	Case–control	UH	Nederland	116 (67/49)	61/68	11.7/11.8	CRRT + CytoSorb	CRRT	NR	47.8%/51.0%
Rugg et al. [2020] [21]	Case–control matched analysis	UH	Austria	84 (42/42)	64/68	13.0/12.0	CRRT + CytoSorb	CRRT	Up to 719 h (median 21.4 h) after ICU admission	21.4%/47.6%
Schittek et al. [2020] [22]	Case–control	TH	Germany	76 (43/33)	63/62	35.0/39.0	CVVHDF + CytoSorb	CVVHDF	NR	72.1%/66.7%
Wendel Garcia et al. [2021] [23]	Case–control matched analysis	UH	Switzerland	96 (48/48)	58/57	14.0/14.0	CytoSorb for 3 consecutive 24 h sessions	Standard medical care	Within 24 h of diagnosis	67.0%/42.0%
Stockmann et al. [2022] [24]	Open-label, RCT	UH	Germany	49 (23/26)	61/66	14.0/14.0	CRRT + CytoSorb for 3 to 7 24 h sessions	CRRT	After diagnosis of septic shock	78.0%/73.0%
Jakopin et al. [2023] [25]	Case–control	TH	Slovenia	102 (44/58)	64/70	NR	CVVH + CytoSorb	CVVH	NR	50.0%/65.5%
Mariano et al. [2024] [26]	Case–control	UT	Italy	35 (11/24)	63/72	12/12	CRRT + CytoSorb	CRRT	After 72 h of diagnosis of sepsis	45.4%/70.8%
Nikumbhe et al. [2024] [27]	Case–control	TH	India	40 (20/20)	45/51	10.9/11.6	CRRT/SLED + CytoSorb	Standard medical care + CRRT/SLED	Not reported	70.0%/75.0%

Legend: CS—CytoSorb; C—control; TH—tertiary hospital; UH—university hospital; SOFA—Sequential Organ Failure Assessment; APACHE II—acute physiology and chronic health evaluation II; RCT—randomized control trial; CRRT—continuous renal replacement therapy; CVVHDF—continuous veno-venous hemodiafiltration; CVVH—continuous veno-venous hemofiltration; NA—not available.
